# Pirrie’s Surgery

**Published:** 1852-08

**Authors:** 


### PIRRIE'S SURGERY.

We received a copy of this excellent treatise too late for notice in this number. In glancing over it, it appears to be admirably executed and well calculated for a text book for students, amongst whom, we doubt not, it will soon become popular.

